# An analysis of the global burden of gallbladder and biliary tract cancer attributable to high BMI in 204 countries and territories: 1990–2021

**DOI:** 10.3389/fnut.2024.1521770

**Published:** 2024-12-16

**Authors:** Zhuowen Hu, Xue Wang, Xin Zhang, Wuping Sun, Jun Mao

**Affiliations:** Department of Clinical Laboratory, The Fifth Affiliated Hospital, Southern Medical University, Guangzhou, Guangdong, China

**Keywords:** gallbladder and biliary tract cancer, global burden, high BMI, health inequalities, mortality

## Abstract

**Background:**

Gallbladder and biliary tract cancers (GBTCs) are aggressive with poor prognosis, often undetected until advanced stages. High Body Mass Index (BMI) is a significant risk factor, contributing substantially to GBTC mortality and Disability-Adjusted Life Years (DALYs). This study aimed to quantify the global burdens of GBTCs attributable to high BMI from 1990 to 2021, thereby developing more rational prevention and treatment strategies for GBTC.

**Methods:**

Data were extracted from the Global Burden of Disease (GBD) 2021. Age-standardized rates of mortality (ASMR), and DALYs (ASDR) for GBTCs due to high BMI were calculated by years, genders, ages, geographical locations, and sociodemographic index (SDI). The estimated annual percentage change (EAPC) was calculated to evaluate the temporal trends from 1990 to 2021. Decomposition and frontier analyses were conducted to understand the driving forces behind burden changes and to identify top-performing countries. Inequality analysis was conducted to assess burden disparities across different SDI levels. The disease burden was forecasted through 2035 using the Bayesian age period cohort (BAPC) model.

**Results:**

Globally, ASMR and ASDR for GBTCs related to high BMI decreased from 1990 to 2021; however, the absolute number of deaths and DALYs cases more than doubled, and similar patterns are projected to continue over the next 14 years in the absence of intervention. High SDI regions showed higher burdens due to higher obesity rates, population growth, and aging, while low SDI regions faced higher EAPCs due to limited resources. Moreover, this inequality has become more significant. Females were more susceptible across all age groups. Notable variations in burden management were observed among countries, with some low SDI nations demonstrating superior performance to high SDI countries.

**Conclusion:**

Despite the decline in rates, the burden of GBTCs attributable to high BMI remains substantial, underscoring the need for targeted prevention strategies for high BMI, particularly in high SDI regions. Gender and age disparities necessitate tailored health interventions.

## 1 Introduction

Gallbladder and biliary tract cancers (GBTCs) are aggressive malignancies, generally characterized by a poor prognosis, encompassing gallbladder cancer (GBC), intrahepatic cholangiocarcinoma (ICC), extrahepatic cholangiocarcinoma (ECC), and ampulla of Vater cancer (AVC) (1). The early stages of GBTCs often go unnoticed due to a lack of distinctive symptoms, leading to delayed diagnosis and a high likelihood of advanced disease at the time of detection. Radical surgical resection is the sole potentially curative treatment (2). Unfortunately, it is estimated that less than 30% of patients receive curative surgical resection (3), and a 5-year overall survival (OS) rate of less than 10% (4). In 2019, there were 199,211 incident cases and 172,441 deaths globally, representing increases of 84.8% and 81.8% from 1990, respectively (5). Furthermore, the epidemiology of GBTCs is influenced by many factors, such as geographic location, gender, and age. Countries in the Asia-Pacific region and South America exhibited higher incidence rates compared to those in European and North American countries (6–8). Women and the elderly exhibit a higher susceptibility to these diseases (7). The reasons for this disparity are multifaceted, encompassing differences in dietary habits (9–11), the prevalence of obesity (12–15), and socioeconomic status (16–18).

With a burgeoning corpus of research indicating that elevated Body Mass Index (BMI) can foster tumorigenesis via chronic inflammation (19, 20), hormonal dysregulation (21), and augmented energy provision to malignant cells. For example, obesity is characterized by elevated levels of the orexigenic hormone ghrelin, which is produced in the gastric fundus and is associated with tumor development (22). This highlights the complex interplay between obesity, hormonal imbalances, and cancer progression, underscoring the need for further investigation into these mechanisms to inform prevention and treatment strategies. The relationship between high BMI and GBTCs is well-established, with a considerable portion of GBTCs attributable to modifiable risk factors, particularly high BMI (14, 23, 24). High BMI is a predominant risk factor, responsible for a significant proportion of GBTC-related deaths and Disability-Adjusted Life Years (DALYs) worldwide (5, 25). Consequently, comprehending the global burden of GBTCs, particularly that attributable to high BMI, is essential for policymakers to efficiently allocate resources and devise targeted prevention strategies.

The Global Burden of Disease (GBD) 2021 Study offers an exhaustive dataset to assess the burden of diseases and injuries, encompassing those related to GBTCs. Prior research has predominantly concentrated on the overall burden of GBTCs (5, 25), yet the burden specifically attributable to high BMI remains ill-defined. This analysis seeks to quantify the global, regional, and national burden of mortality and DALYs due to GBTCs, with a focus on high BMI as a risk factor. By analyzing temporal trends in disease burden, we can pinpoint regions experiencing the most substantial increases, thereby informing cancer control and prevention strategies. This study will also investigate the correlation between the burden of GBTCs and socioeconomic indices, such as the Sociodemographic Index (SDI) and the Human Development Index (HDI), to evaluate the influence of socioeconomic factors on health outcomes. Furthermore, the study aims to assess the contribution of aging, population, and epidemiological factors to GBTCs attributable to high BMI, analyze inequalities among countries, and project changes through 2035. The findings from this analysis will be pivotal in informing public health policies and initiatives aimed at reducing the burden of GBTCs related to high BMI.

## 2 Methods

### 2.1 Data source

For this investigation, we extracted data from the GBD 2021, which offers a comprehensive and systematic assessment of the global, regional, and national disease burden due to various risk factors (26), including high BMI, and its attributable impact on specific diseases such as GBTCs. The GBD data encompasses a wide array of indicators such as incidence, prevalence, mortality, and DALYs for 204 countries and territories from 1990 to 2021 (27). We downloaded the data from 1990 to 2021 via the Global Health Data Exchange query tool.^1^ We Selected “High Body Mass Index” in the Risk list and “Gallbladder and biliary tract cancer” in the Cause list. We collected data on the absolute numbers and age-standardized rates (ASR) of Deaths and DALYs from 21 regions and 204 countries for GBTCs attributable to high BMI. We also obtained data on the SDI^2^ and HDI^3^ for each country.

### 2.2 Definitions

The diagnosis of GBTC is typically based on (1) Imaging studies, including ultrasound, CT, MRI, and PET, are used for identifying suspicious space-occupying lesions or tumors; (2) Serum tumor markers, including CA199, which may be elevated in patients with cholangiocarcinoma and can be used alongside other diagnostic tests for a comprehensive diagnosis; (3) Pathological confirmation, considered the gold standard, involves biopsy or surgical resection and subsequent histopathological examination of tissue samples to confirm the diagnosis and determine the grade and stage of cancer; additionally, several auxiliary diagnostic methods include Endoscopic Retrograde Cholangiopancreatography (ERCP), Intraductal Ultrasound (IDUS), and Cholangioscopy (28). GBTCs are identified using the International Classification of Diseases 10th revision (ICD-10) codes C23, C24–C24.9. High BMI classification applies to individuals aged 20 years or older with a BMI exceeding 25 kg/m^2^ (26). The SDI, as formulated by GBD researchers, includes three key indicators: fertility rates for individuals under 25 years, educational attainment for those aged 15 and above, and *per capita* income. The SDI, calculated using the geometric mean and scaled from 0 to 1, classifies regions and nations into five distinct groups: low (<0.46), low-middle (0.46–0.60), middle (0.61–0.69), high-middle (0.70–0.81), and high SDI (>0.81). The HDI, as defined by the United Nations Development Program, is a standard tool for assessing a country’s human development. It evaluates a country’s progress across life expectancy, education, and literacy rates, and the standard of living and income. The HDI categorizes countries into four tiers based on their development status: very high (0.8–1.0), high (0.7–0.79), medium (0.55–0.70), and low (<0.55).

### 2.3 Statistical analysis

#### 2.3.1 Burden description

The primary metrics for analysis were the age-standardized mortality rate (ASMR) and the age-standardized DALY rate (ASDR) for GBTCs attributable to high BMI. These metrics were used to describe the burden of regions and nations. We also calculated the percentage change (PC) and estimated annual percentage change (EAPC) to quantify the trends in these rates from 1990 to 2021. The EAPC was calculated using a linear regression model accounting for yearly changes, offering an overview of increasing or decreasing trends over time. The computation of the EAPC has been detailed previously (29).

#### 2.3.2 Decomposition analysis

We employed decomposition analysis using Das Gupta’s method (30) to examine the contributions of various factors, including age structure, epidemiological changes, and population growth, to the overall change in Deaths and DALYs. This method enhances our understanding of the driving forces behind the observed changes in the burden of GBTCs attributable to high BMI.

#### 2.3.3 Frontier analysis

Frontier analysis was employed to establish optimal benchmarks for the burden of GBTCs attributable to high BMI by comparing the performance of various countries and territories with the highest-achieving ones. This approach identifies top-performing nations and positions them as role models for others. For each country and territory, we calculated the “effective difference,” representing the gap between the current burden of GBTCs attributable to high BMI and the potential burden achievable given their SDI.

#### 2.3.4 Cross-country inequalities analysis

To quantify the cross-country inequalities in the burden of GBTCs attributable to high BMI across 204 countries and territories from 1990 to 2021, we employed the slope index of inequality (SII) and the concentration index (CI) (31). These metrics are standard indicators recommended by the World Health Organization (WHO) to measure absolute and relative inequality in health outcomes. The SII calculation involved regressing the DALYs or death rate in all age populations for each country due to high BMI-related GBTCs on the SDI-associated relative position scale, which was defined by the population’s midpoint in a cumulative distribution ranked by SDI. The CI calculation entailed fitting a Lorenz curve to the observed cumulative relative distribution of the population ranked by SDI and the corresponding cumulative fraction of DALYs or deaths attributed to high BMI (32). Both indicators measure the degree to which the burden of disease is distributed across countries of different sociodemographic levels, with higher values indicating greater inequality.

#### 2.3.5 Predictive analysis

To forecast the deaths of GBTCs attributable to high BMI from 2022 to 2035 with no intervention, we employed the Bayesian age-period-cohort (BAPC) model, which integrates nested Laplace approximations (33). This model is capable of managing age-stratified cancer incidence and mortality rates and is particularly useful for projecting future trends amidst significant demographic shifts.

#### 2.3.6 Statistics

The ASMR and ASDR for GBTCs attributable to high BMI were calculated and presented per 100,000 population, along with their corresponding 95% uncertainty intervals (UIs). These metrics provide a standardized measure of the disease burden, allowing for comparisons across different countries and periods. All statistical analyses and data visualizations in this study utilized the Health Equity Assessment Toolkit developed by the WHO and R software (version 4.4.1).

## 3 Results

### 3.1 Global and regional burden of GBTCs attributable to high BMI

**Figure 1:**
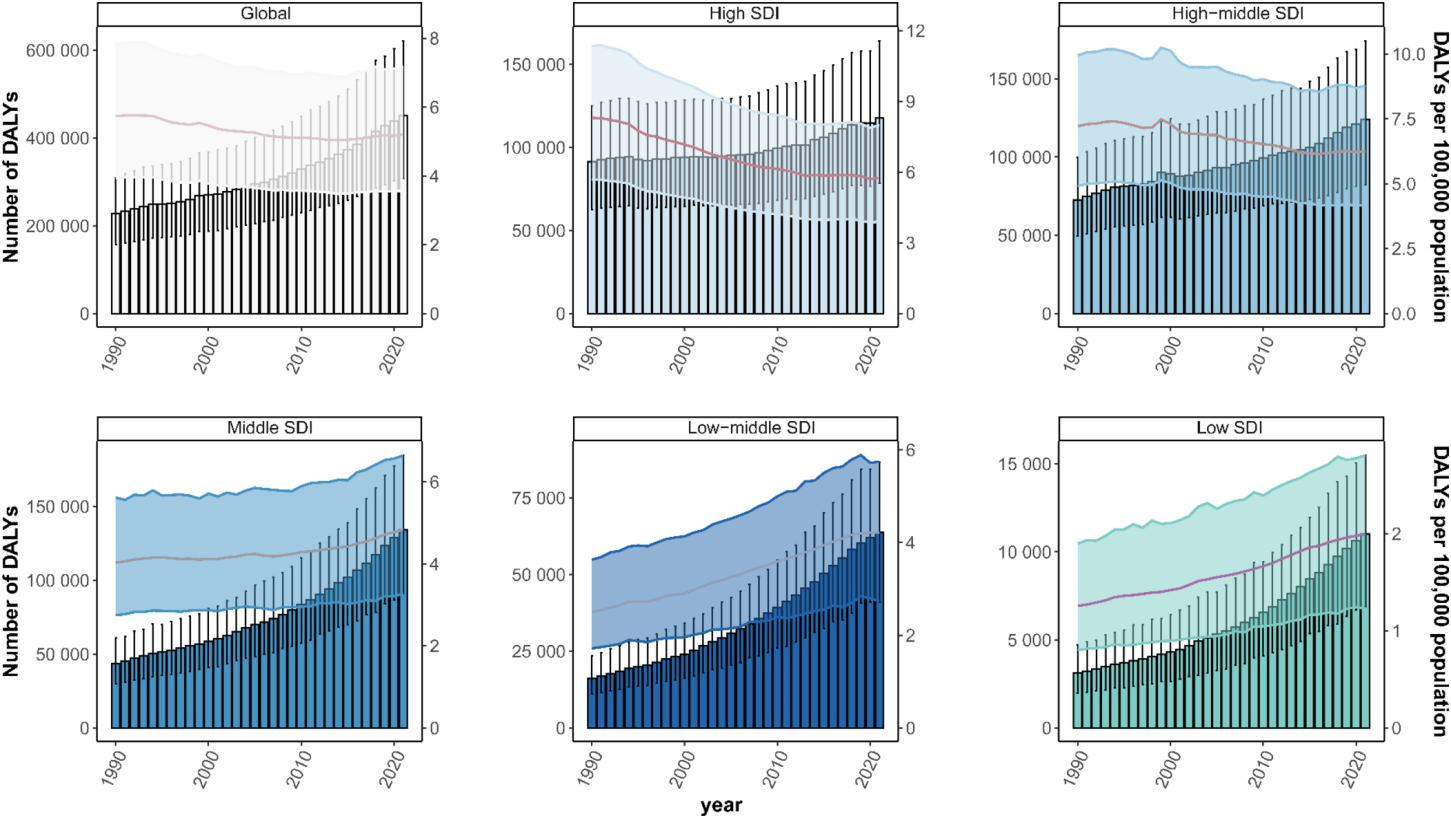
Disability-Adjusted Life Years (DALYs) cases and ASDR of GBTCs attributable to high BMI from 1990 to 2021. DALYs, disability-adjusted life years; ASDR, age-standardized DALY rate; GBTCs, gallbladder and biliary tract cancers.

At the global level, the number of death cases increased from 9,680 (95%UI: 6644–13,261) in 1990 to 20,102 (95%UI: 13656–27,792) in 2021 (Table 1), and the number of DALYs cases increased from 227,609 (95%UI: 156891–309,773) to 451,489 (95%UI: 308188–621,591) in 2021 (Table 2). However, the ASMR and ASDR were 0.237 (95%UI: 0.161–0.328) and 5.203 (95%UI: 3.560–7.169) per 100,000 population in 2021, which decreased by 9.853% (2.419–18.351%) and 9.317% (0.993–19.521%) between 1990 and 2021, respectively. Temporal trend analysis revealed a reduction in the ASMR and ASDR for GBTCs related to high BMI, with EAPCs of −0.46 (95%CI: −0.53 to −0.38) and − 0.44 (95%CI: −0.52 to −0.36), respectively (Tables 1, 2; Figure 1; Supplementary Figure S1).

**Table 1:** Deaths and ASMR of GBTCs attributable to the high BMI in 1990 and 2021 and the PC and EAPC from 1990 to 2021.

Deaths	1990		2021		PC (1990–2021)	EAPC (1990–2021)
	Death cases	ASMR (per 100,000)	Death cases	ASMR (per 100,000)	ASMR	ASMR
Location	No. (95%UI)	No. (95%UI)	No. (95%UI)	No. (95%UI)	No.	No. (95%CI)
Global	9679.890 (6644.007–13261.093)	0.263 (0.180–0.361)	20101.942 (13655.966–27792.118)	0.237 (0.161–0.328)	−0.099 (−0.184 to −0.024)	−0.46 (−0.53 to −0.38)
High SDI	4303.533 (2946.114–5938.132)	0.383 (0.262–0.528)	6305.026 (4113.256–8899.940)	0.279 (0.184–0.392)	−0.272 (−0.328 to −0.223)	−1.15 (−1.25 to −1.05)
High-middle SDI	3038.835 (2073.018–4191.801)	0.321 (0.219–0.443)	5588.582 (3715.910–7895.772)	0.282 (0.187–0.398)	−0.122 (−0.222 to −0.037)	−0.62 (−0.70 to −0.55)
Middle SDI	1621.587 (1106.371–2259.525)	0.169 (0.116–0.235)	5396.141 (3639.267–7418.106)	0.206 (0.139–0.283)	0.213 (0.035–0.376)	0.52 (0.44–0.60)
Low-middle SDI	587.662 (403.664–851.260)	0.103 (0.071–0.148)	2395.981 (1567.947–3267.012)	0.172 (0.114–0.236)	0.679 (0.406–0.944)	1.83 (1.78–1.88)
Low SDI	107.572 (68.734–161.739)	0.049 (0.031–0.073)	393.197 (241.397–553.784)	0.081 (0.049–0.114)	0.664 (0.281–1.126)	1.76 (1.69–1.83)
High-income Asia Pacific	1122.681 (782.578–1542.802)	0.575 (0.398–0.792)	2247.956 (1436.079–3152.358)	0.399 (0.262–0.553)	−0.306 (−0.384 to −0.211)	−1.36 (−1.41 to −1.32)
High-income North America	742.384 (499.391–1033.466)	0.206 (0.139–0.287)	1156.794 (774.536–1575.977)	0.171 (0.116–0.233)	−0.168 (−0.227 to −0.123)	−0.62 (−0.70 to −0.53)
Western Europe	2339.215 (1580.274–3241.904)	0.390 (0.264–0.540)	2450.800 (1624.405–3412.733)	0.239 (0.159–0.331)	−0.388 (−0.424 to −0.356)	−1.70 (−1.86 to −1.53)
Australasia	54.579 (37.415–75.769)	0.232 (0.159–0.322)	111.960 (74.325–156.053)	0.198 (0.131–0.275)	−0.149 (−0.223 to −0.075)	−0.69 (−0.82 to −0.57)
Andean Latin America	121.389 (78.584–174.789)	0.610 (0.396–0.877)	370.768 (231.671–540.249)	0.635 (0.397–0.924)	0.042 (−0.171–0.277)	−0.07 (−0.22–0.08)
Tropical Latin America	353.205 (241.852–487.910)	0.408 (0.279–0.560)	960.821 (651.395–1324.776)	0.376 (0.254–0.520)	−0.077 (−0.149 to −0.002)	−0.41 (−0.55 to −0.28)
Central Latin America	490.448 (339.867–672.718)	0.617 (0.428–0.849)	961.479 (642.083–1339.440)	0.388 (0.259–0.540)	−0.372 (−0.445 to −0.298)	−1.86 (−2.05 to −1.67)
Southern Latin America	606.258 (409.574–826.926)	1.329 (0.899–1.814)	755.240 (508.936–1059.942)	0.857 (0.578–1.200)	−0.355 (−0.398 to −0.307)	−1.50 (−1.60 to −1.41)
Caribbean	43.577 (29.788–58.804)	0.171 (0.117–0.231)	73.503 (50.987–100.555)	0.136 (0.094–0.186)	−0.204 (−0.307 to −0.084)	−0.97 (−1.10 to −0.83)
Central Europe	1038.933 (710.176–1402.881)	0.705 (0.481–0.950)	1069.304 (724.022–1485.252)	0.462 (0.313–0.641)	−0.345 (−0.400 to −0.290)	−1.60 (−1.67 to −1.52)
Eastern Europe	624.414 (426.141–838.685)	0.223 (0.152–0.300)	890.242 (595.668–1225.954)	0.249 (0.167–0.343)	0.115 (−0.019–0.242)	−0.12 (−0.39–0.15)
Central Asia	71.721 (47.800–100.164)	0.157 (0.105–0.220)	112.525 (75.882–156.100)	0.144 (0.097–0.199)	−0.083 (−0.234–0.081)	−0.78 (−1.24 to −0.32)
North Africa and Middle East	311.268 (198.037–456.556)	0.199 (0.126–0.293)	1112.927 (698.740–1587.773)	0.265 (0.165–0.379)	0.332 (0.127–0.576)	1.13 (1.01–1.26)
South Asia	403.010 (270.578–578.823)	0.072 (0.049–0.104)	2408.894 (1418.873–3305.269)	0.166 (0.098–0.227)	1.301 (0.695–1.877)	2.86 (2.79–2.92)
Southeast Asia	213.993 (136.322–304.286)	0.086 (0.055–0.124)	970.343 (608.504–1380.299)	0.154 (0.097–0.220)	0.784 (0.332–1.354)	1.73 (1.67–1.79)
East Asia	1067.600 (713.420–1506.607)	0.134 (0.087–0.189)	4201.251 (2408.063–6246.934)	0.195 (0.111–0.290)	0.460 (0.084–0.900)	1.18 (1.09–1.28)
Oceania	2.080 (1.218–3.062)	0.073 (0.043–0.107)	5.457 (3.263–7.870)	0.075 (0.045–0.106)	0.025 (−0.142–0.219)	0.10 (0.06–0.14)
Western Sub-Saharan Africa	3.080 (2.122–4.453)	0.004 (0.003–0.005)	11.447 (6.637–16.504)	0.007 (0.004–0.009)	0.770 (0.238–1.250)	2.41 (2.04–2.78)
Eastern Sub-Saharan Africa	42.110 (25.658–62.078)	0.058 (0.036–0.086)	125.555 (75.227–184.016)	0.080 (0.048–0.117)	0.365 (0.068–0.789)	0.91 (0.81–1.02)
Central Sub-Saharan Africa	3.973 (2.431–6.264)	0.019 (0.012–0.030)	17.340 (10.063–26.885)	0.034 (0.020–0.052)	0.822 (0.318–1.534)	2.07 (1.91–2.22)
Southern Sub-Saharan Africa	23.973 (14.751–34.748)	0.093 (0.057–0.137)	87.335 (52.620–125.135)	0.161 (0.097–0.231)	0.729 (0.356–1.037)	1.97 (1.78–2.16)

No., number; ASMR, age-standardized mortality rate; UI, uncertainty interval; PC, percentage change; EAPC, estimated annual percentage change; CI, confidential interval; SDI, sociodemographic index.

**Table 2:** DALYs and ASDR of GBTCs attributable to the high BMI in 1990 and 2021 and the PC and EAPC from 1990 to 2021.

DALYs	1990		2021		PC (1990–2021)	EAPC (1990–2021)
	DALYs Cases	ASDR (per 100,000)	DALYs Cases	ASDR (per 100,000)	ASDR	ASDR
Location	No. (95%UI)	No. (95%UI)	No. (95%UI)	No. (95%UI)	No.	No. (95%CI)
Global	227609.123 (156891.312–309772.821)	5.738 (3.946–7.830)	451489.253 (308188.375–621590.880)	5.203 (3.560–7.169)	−0.093 (−0.195 to −0.010)	−0.44 (−0.52 to −0.36)
High SDI	91377.464 (62466.322–125036.996)	8.300 (5.675–11.356)	117789.191 (78610.421–164226.930)	5.758 (3.903–7.994)	−0.306 (−0.355 to −0.262)	−1.31 (−1.42 to −1.21)
High-middle SDI	72606.921 (49385.546–99914.162)	7.221 (4.918–9.948)	124087.576 (82526.145–174299.623)	6.251 (4.154–8.783)	−0.134 (−0.243 to −0.041)	−0.68 (−0.76 to −0.61)
Middle SDI	43794.383 (29828.761–61131.313)	4.032 (2.748–5.616)	134389.408 (90792.926–184807.125)	4.837 (3.272–6.643)	0.200 (0.017–0.373)	0.47 (0.39–0.55)
Low-middle SDI	16242.460 (11079.292–23477.710)	2.507 (1.717–3.631)	63718.356 (40918.976–86689.877)	4.218 (2.729–5.746)	0.682 (0.401–0.954)	1.82 (1.78–1.87)
Low SDI	3115.682 (1970.209–4722.615)	1.256 (0.800–1.897)	11010.945 (6894.530–15495.028)	1.996 (1.233–2.807)	0.589 (0.230–1.053)	1.56 (1.51–1.61)
High-income Asia Pacific	24852.351 (17247.785–33977.854)	12.214 (8.485–16.717)	35488.728 (23529.833–49217.699)	7.506 (4.969–10.295)	−0.385 (−0.452 to −0.296)	−1.77 (−1.83 to −1.71)
High-income North America	15774.664 (10711.790–21928.048)	4.577 (3.114–6.354)	24566.799 (16706.990–33248.507)	3.895 (2.653–5.250)	−0.149 (−0.209 to −0.103)	−0.53 (−0.62 to −0.44)
Western Europe	47043.464 (31826.957–64929.037)	8.184 (5.536–11.304)	44160.965 (29563.495–61183.412)	4.870 (3.288–6.706)	−0.405 (−0.438 to −0.379)	−1.79 (−1.94 to −1.63)
Australasia	1200.950 (821.129–1649.017)	5.139 (3.517–7.056)	2204.911 (1474.253–3084.156)	4.262 (2.881–5.935)	−0.171 (−0.236 to −0.104)	−0.80 (−0.93 to −0.66)
Andean Latin America	3215.850 (2065.840–4662.338)	15.122 (9.737–21.905)	9085.055 (5618.718–13402.600)	15.151 (9.379–22.266)	0.002 (−0.202–0.239)	−0.23 (−0.38 to −0.07)
Tropical Latin America	9262.508 (6318.845–12812.964)	9.714 (6.640–13.408)	23476.755 (15892.037–32063.481)	8.978 (6.088–12.269)	−0.076 (−0.142 to −0.009)	−0.46 (−0.62 to −0.31)
Central Latin America	12854.863 (8846.989–17602.917)	14.842 (10.235–20.335)	23923.618 (15886.814–33520.753)	9.332 (6.201–13.063)	−0.371 (−0.446 to −0.294)	−1.89 (−2.11 to −1.68)
Southern Latin America	14611.786 (9841.009–19989.419)	31.304 (21.100–42.885)	17179.192 (11624.115–23684.398)	20.085 (13.604–27.585)	−0.358 (−0.402 to −0.310)	−1.53 (−1.65 to −1.41)
Caribbean	1106.085 (751.730–1483.255)	4.189 (2.847–5.618)	1806.997 (1234.572–2495.276)	3.357 (2.291–4.640)	−0.199 (−0.306 to −0.077)	−0.94 (−1.07 to −0.80)
Central Europe	23557.717 (16066.968–31940.323)	15.486 (10.557–20.966)	22011.705 (14935.464–30538.755)	10.033 (6.809–13.886)	−0.352 (−0.406 to −0.295)	−1.59 (−1.64 to −1.53)
Eastern Europe	15213.343 (10350.132–20399.559)	5.326 (3.615–7.144)	19895.088 (13252.083–27358.024)	5.682 (3.796–7.798)	0.067 (−0.061–0.192)	−0.30 (−0.59 to −0.02)
Central Asia	1878.281 (1255.518–2624.126)	3.907 (2.611–5.464)	2913.719 (1957.319–4030.893)	3.407 (2.297–4.711)	−0.128 (−0.268–0.026)	−1.00 (−1.45 to −0.55)
North Africa and Middle East	8426.960 (5409.711–12430.105)	4.742 (3.021–6.962)	28624.175 (18052.803–40883.296)	6.014 (3.789–8.585)	0.268 (0.071–0.517)	0.92 (0.81–1.03)
South Asia	11746.993 (7711.826–16846.244)	1.853 (1.236–2.658)	65410.960 (38512.258–89114.657)	4.183 (2.459–5.703)	1.257 (0.661–1.799)	2.79 (2.73–2.84)
Southeast Asia	6084.904 (3856.014–8617.445)	2.203 (1.400–3.127)	25094.244 (15859.275–35285.313)	3.638 (2.291–5.136)	0.652 (0.243–1.189)	1.48 (1.40–1.56)
East Asia	28666.529 (19085.558–40269.987)	3.155 (2.102–4.447)	98907.136 (56555.529–147055.656)	4.453 (2.540–6.631)	0.411 (0.027–0.843)	1.09 (1.02–1.17)
Oceania	63.264 (36.460–94.017)	1.894 (1.107–2.790)	162.349 (96.130–235.817)	1.909 (1.143–2.756)	0.008 (−0.164–0.206)	0.04 (−0.00–0.08)
Western Sub-Saharan Africa	83.719 (54.068–119.356)	0.090 (0.060–0.128)	302.267 (172.063–437.965)	0.146 (0.085–0.211)	0.631 (0.193–1.078)	2.09 (1.72–2.46)
Eastern Sub-Saharan Africa	1200.891 (717.466–1785.534)	1.478 (0.899–2.188)	3481.166 (2102.325–5127.954)	1.910 (1.146–2.808)	0.293 (−0.017–0.743)	0.70 (0.59–0.80)
Central Sub-Saharan Africa	112.843 (68.304–179.049)	0.463 (0.284–0.729)	492.829 (286.138–767.598)	0.825 (0.479–1.275)	0.781 (0.275–1.455)	2.00 (1.85–2.15)
Southern Sub-Saharan Africa	651.157 (404.007–943.732)	2.270 (1.394–3.271)	2300.595 (1379.074–3280.539)	3.797 (2.283–5.417)	0.672 (0.326–0.942)	1.93 (1.75–2.11)

No., number; DALYs, disability adjusted life years; ASDR, age-standardized DALY rate; UI, uncertainty interval; PC, percentage change; EAPC, estimated annual percentage change; CI, confidential interval; SDI, socio-demographic index.

At the regional level, in 2021, the highest death and DALYs cases of GBTCs attributable to high BMI were identified in East Asia with 4,201 (95%UI: 2408–6,247) and 98,907 (95%UI: 56556–147,056), respectively. In contrast, Oceania had the lowest death and DALY cases, 5 (95%UI: 3–8) and 162 (95%UI: 96–236), respectively. However, ASMR and ASDR of GBTCs attributable to high BMI were highest in Southern Latin America at 0.857 (95%UI: 0.578–1.200) and 20.085 (95%UI: 13.604–27.585) per 100,000 population. At the same time, the lowest ASMR and ASDR were identified in Western Sub-Saharan Africa with 0.007 (95%UI: 0.004–0.009) and 0.146 (95%UI: 0.085–0.211) per 100,000 population, respectively. From 1990 to 2021, South Asia had the largest increases in ASMR and ASDR, with EAPCs of 2.86 (95%CI: 2.79–2.92) and 2.79 (95%CI: 2.73–2.84), with PCs of 130.095% (69.460–187.656%) and 125.699% (66.066–179.873%). In comparison, the largest decreases in ASMR and ASDR of GBTCs attributable to high BMI were observed in Central Latin America, with EAPCs of −1.86 (95%CI: −2.05 to −1.67) and −1.89 (95%CI: −2.11 to −1.68), with PCs of −37.183% (−44.487% to −29.758%) and −37.122% (−44.645% to −29.416%), respectively (Tables 1, 2). In terms of SDI regions, in 2021, the highest death cases of GBTCs attributable to high BMI were observed in High SDI, which were 6,305 (95%UI: 4113–8,900), and the Middle SDI had the highest number of DALYs cases, 134,389 (95%UI: 90793–184,807). Temporal trend analysis revealed an increase in ASMR and ASDR in Middle SDI, Low-Middle SDI, and Low SDI, but a decrease in High SDI and High-Middle SDI (Tables 1, 2; Figure 1; Supplementary Figure S1).

### 3.2 National burden of GBTCs attributable to high BMI

**Figure 2:**
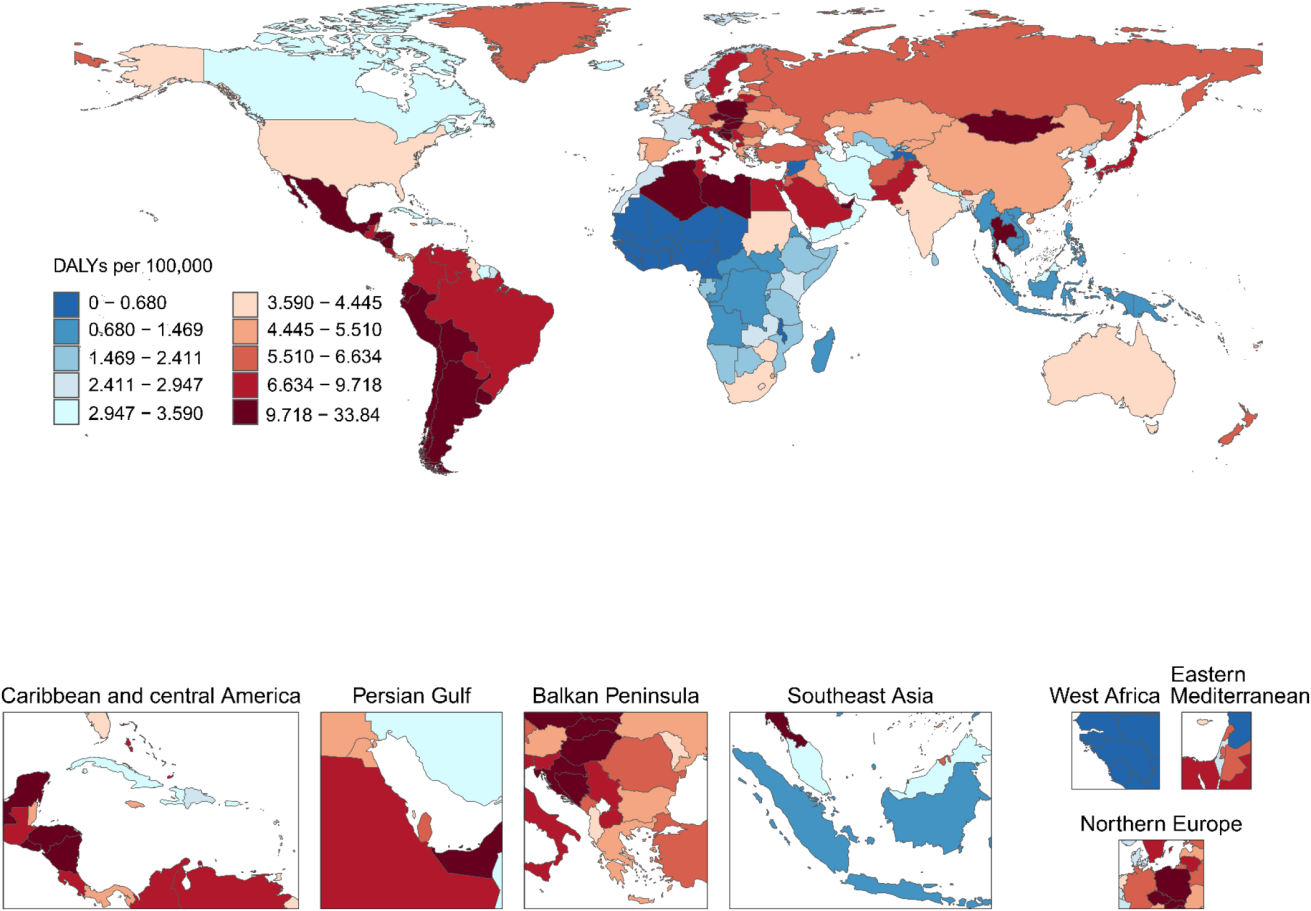
Age-standardized DALY rate (ASDR) of GBTCs attributable to high BMI per 100,000 population in 2021, by country. ASDR, age-standardized DALY rate; GBTCs, gallbladder and biliary tract cancers.

At the national level, China (4,053, 95%UI: 2279–6,034), India (1805, 95%UI: 1069–2,508), Japan (1756, 95%UI: 1105–2,508), and the United States of America (1,032, 95%UI: 691–1,398) had the highest number of death cases in 2021. For GBTCs-related DALYs cases due to high BMI, China, India, Japan, and Brazil were the top four countries, with 95,576 (95%UI: 53908–142,812), 48,287 (95%UI: 28368–66,650), 26,074 (95%UI: 17300–36,694), and 23,042 (95%UI: 15520–31,530), respectively. Additionally, in 2021, ASMR and ASDR of GBTCs attributable to high BMI were highest in Chile at 1.474 (95%UI: 1.000–2.073) and 33.844 (95%UI: 22.973–46.850). Next were the United Arab Emirates, Bolivia (Plurinational State of), and Libya. From 1990 to 2021, Cabo Verde had the largest increases in ASMR and ASDR, with EAPCs of 5.06 (95%CI: 4.35–5.77) and 4.68 (95%CI: 3.96–5.41), with PCs of 377.299% (42.846–718.878%) and 318.027% (27.968–610.641%), respectively. The largest decreases in ASMR and ASDR of GBTCs attributable to high BMI were observed in Turkmenistan, with EAPCs of −4.90 (95%CI: −6.17 to −3.61) and −4.87 (95%CI: −6.13 to −3.59), with PCs of −68.678% (−77.099% to −57.714%) and −68.636% (−77.133% to −57.304%), respectively, and following countries were Sri Lanka, Guatemala, and Bermuda (Figure 2; Supplementary Figure S2; Supplementary Files S1–S4).

### 3.3 GBTCs burden attributable to high BMI by age and sex

**Figure 3:**
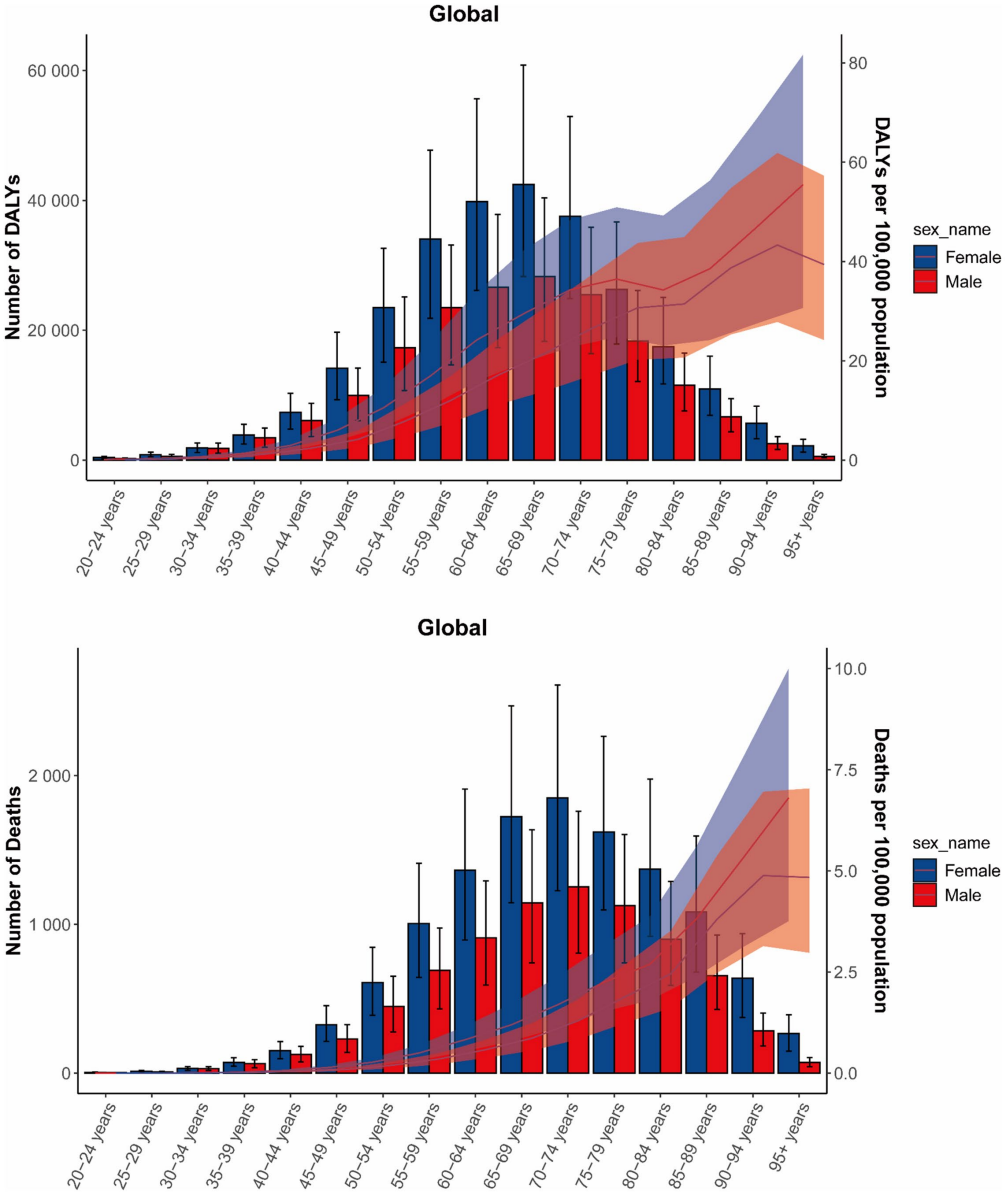
Age-specific numbers and rates of DALYs and deaths of GBTCs attributable to high BMI by age and sex, in 2021. The bars represent the number of GBTC deaths and DALYs attributable to high BMI. The line represents the age-specific death rates and DALYs rates for females and males attributable to high BMI at the global level. The shaded area represents the 95% UI for the rates. DALYs, disability-adjusted life years; GBTCs, gallbladder and biliary tract cancers; UI, uncertainty interval.

Globally, in 2021, the age-specific rates of death and DALYs in females for GBTCs attributable to high BMI increased with increasing age, with the exception of DALYs in the 75–84 age group. In males, the patterns of death and DALYs paralleled those in females, except for individuals aged 90 and over. In 2021, death rates peaked in the 95+ age group, while DALYs rates reached their highest in the 90–94 age group, with both rates being higher in females across all age groups. For both genders, the number of deaths peaked in the 70–74 age group, and the DALYs numbers reached their highest level in those aged 65–69 years. Similarly, the number of deaths and DALYs in females were higher than those in males across all age groups (Figure 3). For SDI regions, the trends of age-specific death rates for both genders in high SDI, high-middle SDI, and middle SDI were consistent with global trends. In the low-middle SDI and low SDI regions, the age-specific death rates increased with increasing age, except in those aged 90+ years. Regarding the DALYs rates in females, high SDI regions increased in all age groups, but middle SDI, low middle SDI, and low SDI regions started to decrease in the 70–74 age group. For all SDI regions, there was a notable trend in the deaths and DALYs from GBTCs linked to high BMI, whereby numbers initially increased and subsequently decreased with age. Regions with higher SDI observed peak death and DALYs numbers at higher ages. Generally, the burdens of GBTCs attributable to high BMI in females were higher than those in males across most age groups in all SDI regions (Supplementary Figures S3, S4).

### 3.4 GBTCs burden attributable to high BMI was associated with SDI and HDI

**Figure 4:**
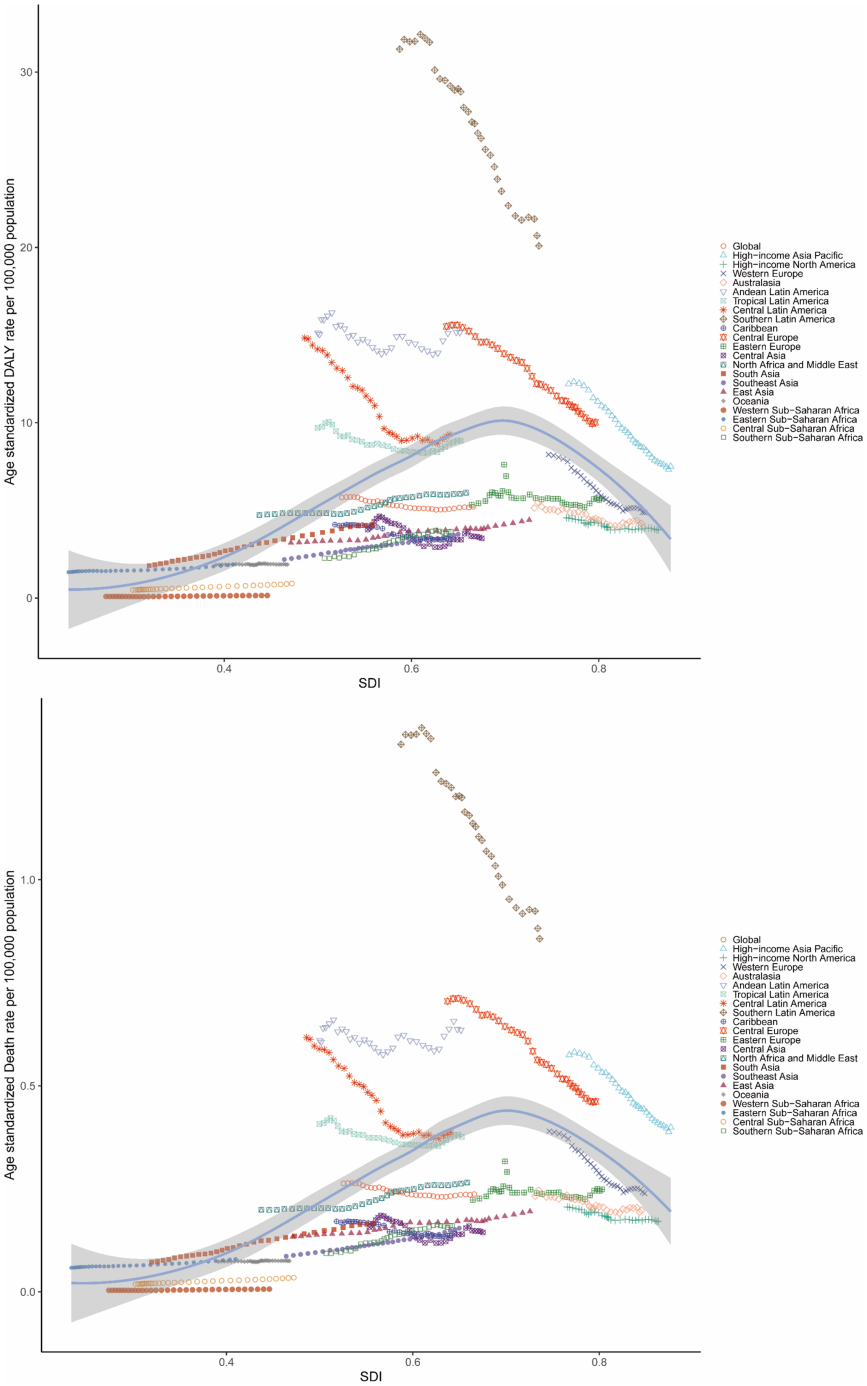
Age-standardized DALY rate and ASMR of GBTCs attributable to high BMI in 21 GBD regions by the SDI, 1990–2021. ASDR, age-standardized DALY rate; ASMR, age-standardized mortality rate; GBTCs, gallbladder and biliary tract cancers; GBD, global burden of disease; SDI, sociodemographic index.

**Figure 5:**
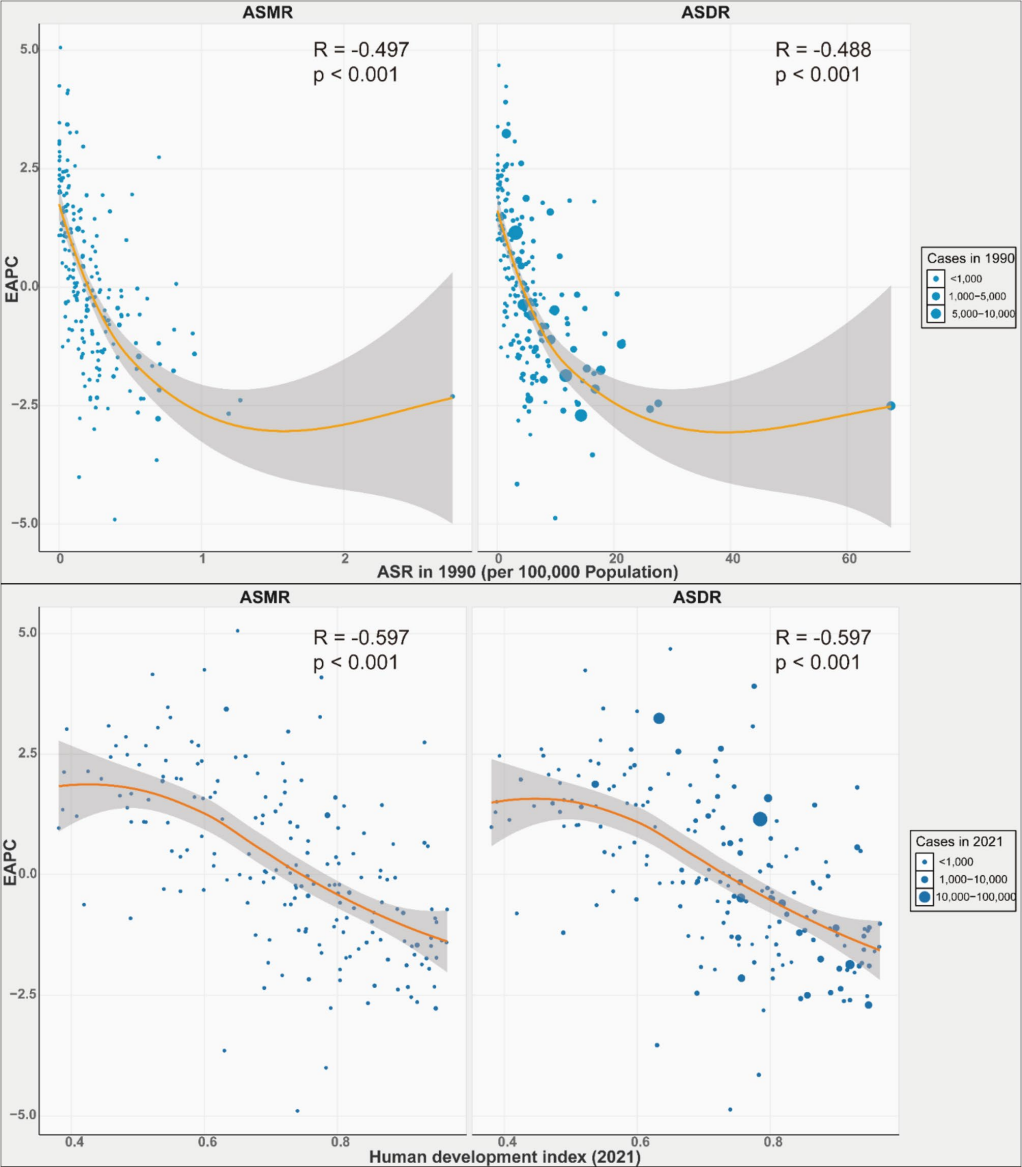
Correlation between EAPCs and ASR of GBTC in 1990, and HDI in 2021. EAPCs, estimated annual percentage changes; ASR, age-standardized rate; GBTC, gallbladder and biliary tract cancer; HDI, human development index.

From 1990 to 2021, the ASMR and ASDR for GBTCs attributable to high BMI rose with increasing SDI, peaking when SDI reached approximately 0.7, after which the rates declined. Notably, Andean Latin America, Central Europe, Southern Latin America, and High-income Asia Pacific exhibited ASMRs and ASDRs higher than expected for their development levels (Figure 4). Nationally, the correlation between ASMR and ASDR for GBTCs attributable to high BMI and SDI mirrored the regional pattern, increasing until SDI reached around 0.8, beyond which the rates started to decline. Chile, Bolivia (Plurinational State of), United Arab Emirates, and Libya had higher than expected burdens (Supplementary Figure S5). A significant negative association (*R* = −0.497, −0.488, *p* < 0.001) was observed between the EAPCs and ASMR or ASDR in 1990 (Figure 5). Furthermore, a strong negative correlation (*R* = −0.597, −0.597, *p* < 0.001) existed between HDI in 2021 and EAPCs of ASMR and ASDR (Figure 5).

### 3.5 Decomposition analysis of GBTCs burden attributable to high BMI

**Figure 6:**
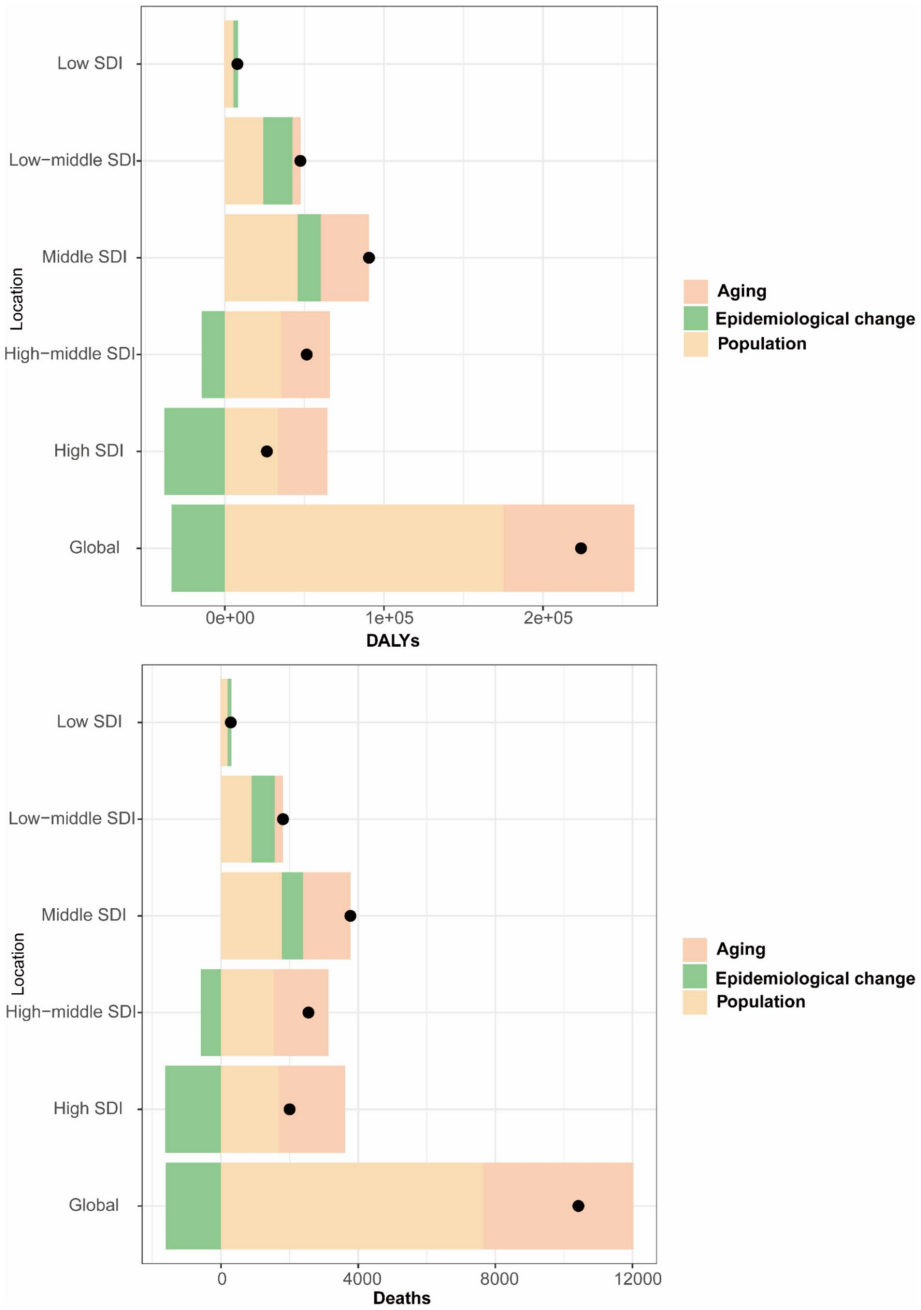
Decomposition analysis of changes in the DALYs and deaths of GBTCs attributable to high BMI between 1990 and 2021 across SDI regions. DALYs, disability-adjusted life years; GBTCs, gallbladder and biliary tract cancers; SDI, socio-demographic index.

We conducted a decomposition analysis to further explore the impact of aging, population, and epidemiological changes on deaths and DALYs of GBTCs attributable to high BMI. Globally, the number of death cases increased by 10422.05 from 1990 to 2021. Population contributed 7636.26 (73.27%), aging contributed 4394.11 (42.16%), and epidemiological change (ASMR change) contributed −1608.31 (−15.43%). In terms of DALYs, between 1990 and 2021, the global increase was 223,880.13, with population contributing 175,099.22 (78.21%), aging contributing 82,203.47 (36.72%), and epidemiological change (ASDR change) contributing −33,422.56 (−14.93%), respectively. In SDI regions, the contributions of aging and population were highest in the high SDI, for deaths, they accounted for 1955.41 (97.7%) and 1672.06 (83.54%), for DALYs, they accounted for 31102.9 (117.76%) and 33367.94 (126.34%), respectively. Low-middle SDI and low SDI had the highest contribution of epidemiological change in death, with 673.01 (37.22%), and 107.71 (37.71%), respectively. For DALYs, the pattern was similar to that of deaths in low-middle and low SDI regions, with contributions of 18,140.18 (38.21%) and 2,847.86 (36.07%), respectively (Figure 6).

### 3.6 Cross-country inequality analysis of GBTCs burden attributable to high BMI

**Figure 7:**
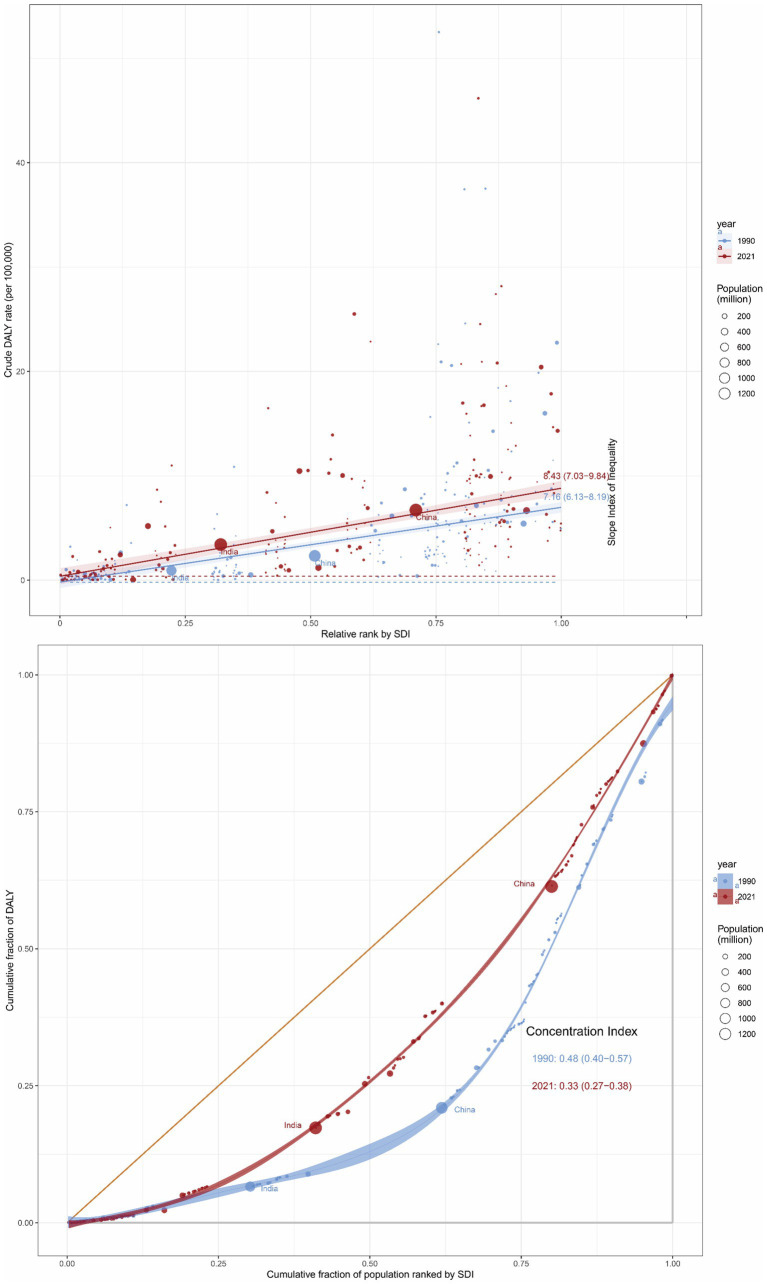
Inequality analysis of DALYs in GBTC attributable to high BMI in 1990 and 2021 across the world. DALYs, disability-adjusted life years; GBTCs, gallbladder and biliary tract cancers.

There were significant relative and absolute SDI-related inequalities in the burden of GBTCs attributable to high BMI, with an increasing trend in SII from 1990 to 2021. Disproportionately higher burdens of GBTCs attributable to high BMI were observed in the higher SDI regions. The SII was 7.16 (95%CI, 6.13–8.19) in 1990 and increased to 8.43 (95%CI, 7.03–9.84) in 2021, indicating a larger gap in the DALYs rate between regions with the highest and lowest SDI (Figure 7). Furthermore, the CI was 0.48 (95%CI, 0.40–0.57) in 1990 and decreased to 0.33 (95%CI, 0.27–0.38) in 2021, suggesting persistent inequalities between low and high SDI regions, though the relative concentration of the burden had lessened (Figure 7). Similarly, for the death rate of GBTCs attributable to high BMI, the SII increased from 0.31 (95%CI, 0.27–0.36) in 1990 to 0.41 (95%CI, 0.35–0.48) in 2021, and the CI was 0.53 (95%CI, 0.44–0.62) in 1990 and decreased to 0.39 (95%CI, 0.33–0.46) in 2021 (Supplementary Figure S6).

### 3.7 Frontier analysis of GBTCs burden attributable to high BMI

**Figure 8:**
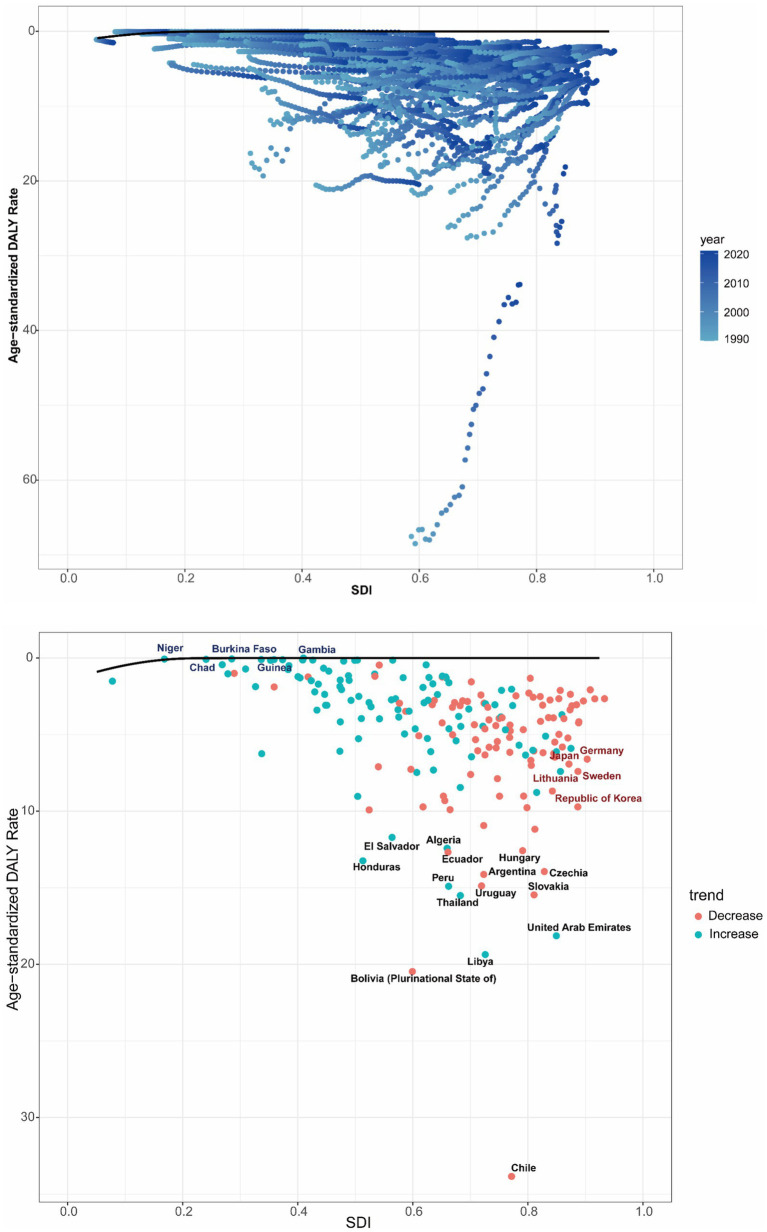
Frontier analysis of ASDR in GBTCs attributable to high BMI based on SDI from 1990 to 2021, and specifically in 2021. ASDR, age-standardized DALY rate; GBTCs, gallbladder and biliary tract cancers; SDI, socio-demographic index.

The frontier analysis was performed to evaluate the possible enhancement room for the burdens of GBTCs attributable to high BMI relative to SDI levels. Taking the SDI of each country into account, the study pinpointed the top 15 countries or regions with the greatest potential for advancement, with an effective difference (ef_df) ranging from 11.71 to 33.84. These countries included Chile (33.84), Bolivia (Plurinational State of) (20.47), Libya (19.36), United Arab Emirates (18.13), Thailand (15.51), Slovakia (15.46), Peru (14.90), Uruguay (14.88), Argentina (14.13), Czechia (13.94), Honduras (13.24), Ecuador (12.68), Hungary (12.58), Algeria (12.42) and El Salvador (11.71). Countries with a low SDI (<0.5) that were on the frontier included Gambia (ef_df: 3.19 × 10^-5), Niger (ef_df: 0.04), Burkina Faso (ef_df: 0.06), Chad (ef_df: 0.07), and Guinea (ef_df: 0.10). Additionally, high SDI (> 0.85) countries with significant potential for improvement relative to their development stage were Germany (ef_df: 6.60), Japan (ef_df: 6.92), Sweden (ef_df: 7.40), Lithuania (ef_df: 7.41), and Republic of Korea (ef_df: 9.72) (Figure 8).

### 3.8 Prediction analysis of GBTCs burden attributable to high BMI

**Figure 9:**
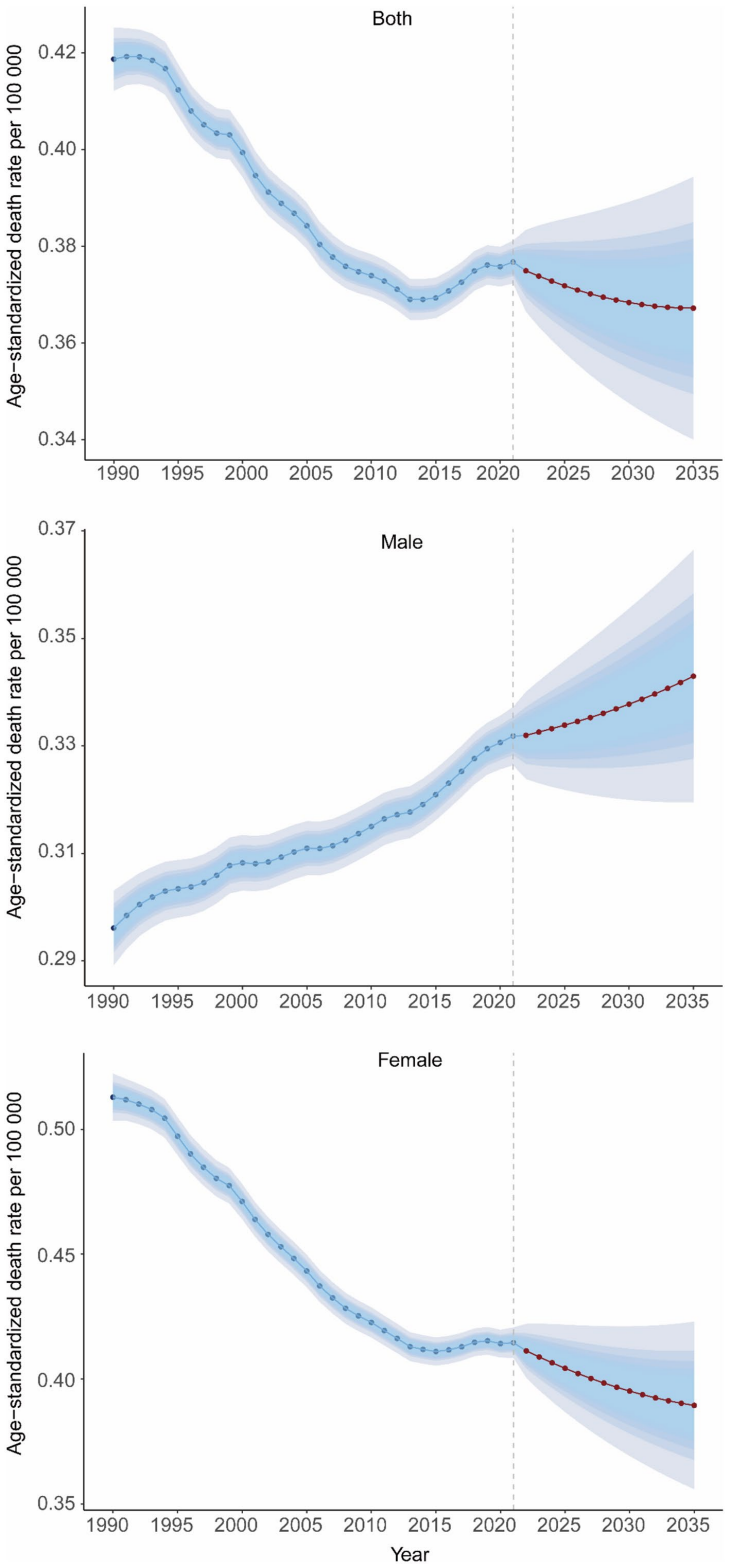
Temporal trends of ASMR in GBTCs attributable to high BMI at the global level from 1990 to 2035 for both genders, males, and females. ASMR, age-standardized mortality rate; GBTC, gallbladder and biliary tract cancers.

The BAPC model analysis was conducted to predict the future burdens of GBTCs attributable to high BMI in the absence of intervention. As shown in Figure 9, the ASMR of GBTCs attributable to high BMI would continue to decrease in the next decade, and it would be approximately 0.37 (95%CI, 0.34–0.39) in 2035 for both genders, globally. The ASMR is projected to increase for males over the next 14 years, reaching 0.34 (95%CI, 0.32–0.37) in 2035. By contrast, the ASMR of GBTCs related to high BMI for females is expected to show a descending trend from 2022 to 2035, with a predicted value of 0.39 (95%CI, 0.36–0.42) in 2035. Furthermore, the study also predicted the age-specific mortality cases and rates for both sexes, male and female. The number of deaths is projected to increase in all age groups for both sexes, males and females from 2022 to 2035. For females and both genders, the mortality rate in the 20–54 age group is expected to increase, but decrease in those aged 65 and older. For males, the mortality rate showed an increasing overall trend from 24 to 84 years (Supplementary Figures S7–S12).

## 4 Discussion

Previous research has demonstrated that a high BMI is a leading risk factor for GBTCs (5, 25), accounting for 15.2% of deaths and 15.7% of DALYs globally in 2019 (5). There is an urgent need to understand the burden of GBTCs attributable to high BMI. The study presents a comprehensive summary of the burdens of GBTCs attributable to high BMI at global, regional, and national levels, and projects burden trends for the next 14 years with no intervention using the latest GBD 2021 data. This analysis aims to provide policymakers with insights for effective resource allocation and the design of targeted prevention strategies.

The results revealed that the ASMR and ASDR for GBTCs associated with high BMI decreased from 1990 to 2021, with EAPCs of −0.46 and −0.44, respectively. However, the absolute cases of deaths and DALYs in 2021 were more than double those in 1990, globally. This trend was closely related to population growth and aging. However, epidemiological change could not offset the contributions of population growth and aging to the increased burdens of GBTCs attributable to high BMI. Specifically, this trend was more frequently observed in regions and nations with relatively high SDI. Regions with high SDI are characterized by longer life expectancies and more rapid aging processes (34, 35). This may partially explain why the highest contributions of population growth and aging to deaths and DALYs in GBTCs related to high BMI were observed in high SDI regions. In contrast, in regions with relatively low SDI, due to insufficient medical resources and limited health services (36), early-stage GBTCs could not be detected timely, and the PCs and EAPCs in ASMR and ASDR were higher, therefore, epidemiological change was also an important driving force for the burdens in low SDI regions.

Although the burdens of GBTCs associated with high BMI are closely related to population growth and aging, other factors must also be considered. The increasing trend of obesity or high BMI in recent years was also an undeniable factor (37, 38). Overall obesity is associated with several comorbidities and increased malignancy from all organ systems. It is associated with endometrial cancer, colorectal cancer, gastroesophageal, hepatocellular and gallbladder cancer mainly through MASLD and gallbladder stones, respectively. Studies have shown that being overweight is closely related to higher gallbladder cancer risks (14, 23). Changes in lifestyle and eating habits are the major factors causing obesity. As living standards improved, people consumed more high-calorie foods, engaged in less physical activity, and maintained more sedentary behaviors, which could increase obesity (39). In this way, facing the severe challenges of population growth and aging globally, it is imperative to implement measures to control the obesity rate. Policymakers should encourage a shift toward balanced eating diets and promote increased physical activity.

The SDI and HDI levels significantly affected the geographical distribution of high BMI-related GBTC burdens. Regions or countries with higher SDI or HDI levels often had higher ASMR and ASDR but lower EAPCs in GBTCs associated with high BMI. The health inequalities in deaths and DALYs were more pronounced between high SDI regions and low SDI regions from 1990 to 2021. This phenomenon is partly attributed to countries with higher SDIs having more advanced diagnostic facilities, complete disease screening mechanisms, and adequate epidemiological data coverage.

Another significant factor is the increase in obesity rates in high SDI regions in recent years. Previous studies suggested that the prevalence of high BMI was significantly associated with nations’ economic status (40, 41). According to the World Obesity Atlas 2024 (42), in high-income countries, the number of overweight and obesity in 2020 was 314.39 and 271.90 millions, respectively, more than sevenfold that in low-income countries. With the development of society’s economy, lifestyles in high SDI countries have changed dramatically in recent years, with an increasing number of people choosing to eat fast food, take more convenient and quicker modes of travel, and use electronic devices more frequently. All of these factors continue to weight gain (43). Therefore, different countries should formulate corresponding strategies according to their actual situations. For high SDI regions, prioritizing obesity prevention, including organizing health campaigns and encouraging healthy eating and increased physical activity, is essential. For low SDI countries, improving residents’ medical insurance and strengthening early screening and diagnosis of diseases are top priorities.

Furthermore, age and gender stratification revealed divergent outcomes; the ASMR of GBTCs related to high BMI increased with age, with higher cases and age-standardized rates observed in females across all age groups. This indicates that a particular demographic requires heightened attention. Previous research has indicated that obesity is more prevalent in women (44, 45), and this demographic generally exhibits a higher incidence of cancer compared to men (46). Despite the study’s projected analysis indicating that the ASMR would decrease for females but increase for males in the next 14 years in the absence of intervention, female deaths are expected to remain higher than males across all age groups. Therefore, it is imperative to allocate additional medical resources to ensure that elderly women receive timely and effective treatment and to implement proactive measures to manage their obesity rates, thereby reducing the burden of GBTCs associated with high BMI in this population.

The frontier analysis revealed substantial variation among countries in managing GBTC burdens attributable to high BMI. Notably, nations with lower SDI, including Gambia, Niger, Burkina Faso, Chad, and Guinea, achieved remarkable success in managing the impact of the burdens. These countries have emerged as models of excellence, demonstrating effective approaches to enhance health outcomes within the constraints of limited resources. Conversely, high-SDI nations such as Germany, Japan, Sweden, Lithuania, and Republic of Korea did not meet expectations in addressing the burden of high BMI-related GBTCs. Geographical factors and dietary practices might contribute to this inconsistency. This situation underscores the urgent need for these high-SDI countries to more effectively optimize and reform the formulation and implementation of health policies.

This study faced several limitations. Firstly, the GBD 2021, while comprehensive, may have insufficient coverage of epidemiological statistics for some low-income countries, potentially leading to an underestimation of the burden of GBTCs attributable to high BMI in these regions. Secondly, high BMI was not classified into subcategories, necessitating further exploration into how varying degrees of high BMI impact GBTC burdens. Lastly, the GBD 2021 database lacked specific classifications for GBTC subtypes, which could obscure differences in burden patterns and impacts of high BMI across subtypes.

## 5 Conclusion

The study reveals that the age-standard rates of death and DALYs in GBTC attributable to high BMI have been declining in recent years, but the actual cases of death and DALYs are increasing globally. Countries and regions with different SDI levels exhibit remarkable disparities in high BMI-related GBTC burdens. Each country must implement strategies tailored to its specific situation. The disparities in GBTC burdens among various genders and age groups underscore the necessity for high BMI prevention and personalized treatment strategies for specific populations. Facing the severe challenges of population growth and aging, public health initiatives must consider these differences, ensuring that resources are allocated wisely and interventions are targeted effectively. To reduce the burden of GBTC, key measures include raising awareness about the negative effects of high BMI, promoting healthy eating patterns and regular physical activity, and enhancing medical care for individuals with obesity.

## Data Availability

The original contributions presented in the study are included in the article/Supplementary material, further inquiries can be directed to the corresponding authors.

## Footnotes

^1^ http://ghdx.healthdata.org/

^2^ https://ghdx.healthdata.org/gbd-2021

^3^ https://hdr.undp.org/data-center/human-development-index#/indicies/HDI
