# Supplementary figures and images for: An analysis of the global burden of gallbladder and biliary tract cancer attributable to high BMI in 204 countries and territories: 1990–2021
Source: Front Nutr. 2024 Dec 16;11:1521770. doi: 10.3389/fnut.2024.1521770 (PMC11729382; doi:10.3389/fnut.2024.1521770)

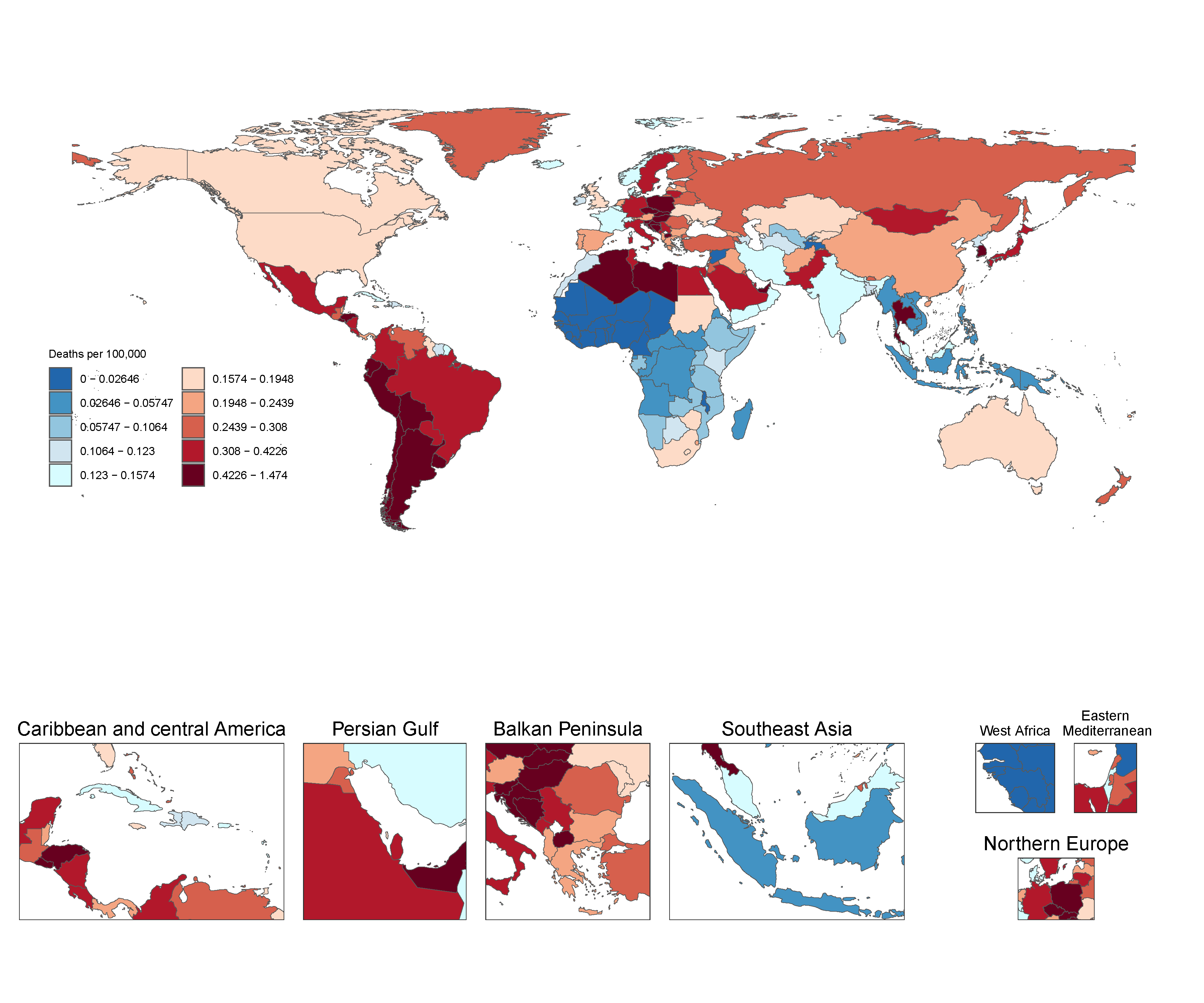

Supplement: Supplementary file 6 [file Image_2.TIF]
